# Oral delivery of bovine tuberculosis vaccine to free-ranging white-tailed deer

**DOI:** 10.3389/fvets.2025.1548627

**Published:** 2025-02-10

**Authors:** Kurt VerCauteren, Abigail Feuka, Michael Lavelle, Michael Glow, Keely Kohen, Patrick Ryan, Tony Aderman, Anthony Duffiney, Mitchell Palmer, Paola M. Boggiatto, Carly Kanipe, Hayden Hamby, Emily Ruell, Melinda Cosgrove, Michael Vanderklok, Nathan Snow, Kim M. Pepin, Henry Campa

**Affiliations:** ^1^United States Department of Agriculture, Animal and Plant Health Inspection Service, Wildlife Services, National Wildlife Research Center, Fort Collins, CO, United States; ^2^United States Department of Agriculture, Animal and Plant Health Inspection Service, Wildlife Services, Gaylord, MI, United States; ^3^United States Department of Agriculture, Agricultural Research Service, Ames, IA, United States; ^4^Michigan Department of Natural Resources, Wildlife Division, Wildlife Health Section, Lansing, MI, United States; ^5^Animal Industry Division, Michigan Department of Agriculture and Rural Development, Lansing, MI, United States; ^6^Department of Fisheries and Wildlife, Michigan State University, East Lansing, MI, United States

**Keywords:** BCG, bovine tuberculosis, disease, *Odocoileus virginianus*, oral, vaccine, white-tailed deer

## Abstract

**Introduction:**

Free-ranging white-tailed deer (*Odocoileus virginianus*) are a self-sustaining reservoir for bovine tuberculosis (bTB) in northeastern lower Michigan, (United States) continually putting the area’s cattle industry at risk. Liberal recreational deer harvest, baiting bans, and mitigation measures on farms have reduced but not eliminated bTB in deer nor have they eliminated transmission to cattle. With apparent prevalence in deer being low (1–2%) but constant, vaccination could be an additional tool to aid in addressing the problem and merits investigation. *Mycobacterium tuberculosis* Bacillus Calmette-Guérin (BCG) vaccine is a widely used human vaccine for tuberculosis that has also been well studied in domestic livestock and wildlife. It is the primary vaccine candidate, and oral delivery is the logical means for delivering it to free-ranging deer, although this has never previously been attempted.

**Materials and methods:**

Building off methods and strategies developed for vaccinating deer, we incorporated BCG vaccine into vaccine delivery units (DUs), consisting of a food-based matrix. We deployed DUs at sites in Michigan with a historically high prevalence of bTB. At each site, 100 DUs were placed systematically 2.5-m apart on 50-m x 10-m grids and monitored with still and video cameras. Consumption, still images, and video data were analyzed to assess uptake of vaccine DUs by deer.

**Results and discussion:**

Vaccine DUs were deployed in 2024 at 11 agricultural sites on private land which had previously demonstrated moderate to high deer activity and at all but two sites >50% of distributed vaccine DU’s were consumed, with 100% consumed at two sites. Deer learned to seek out and consume vaccine DU’s in just 1 to 3 days, with individuals often eating more than the 1 or 2 needed to vaccinate themselves. This high level of consumption was in spite of an exceptionally warm and dry winter, where deer were less food stressed than usual.

## 1 Introduction

Caused by *Mycobacterium bovis*, bovine tuberculosis (bTB) is an infectious disease (1) that is transmitted through direct physical contact and indirect contact via shared feed, water, and fomites (1, 2). bTB persists in numerous wildlife species including European badgers (*Meles meles*) in the United Kingdom (3), France (4), and the Republic of Ireland (5); brushtail possums (*Trichosurus vulpecula*) in New Zealand (6); African buffalo (*Syncerus caffer*) in Southern Africa (7); and wild boar (*Sus scrofa*), red deer (*Cervus elaphus*), and fallow deer (*Dama dama*) in Spain (8, 9). In the United States, free-ranging white-tailed deer (*Odocoileus virginianus*; hereafter referred to as ‘deer’) have been recognized as a reservoir of bTB in the northeastern portion of the lower peninsula of the state of Michigan for over 30 years (10, 11). The disease continually impacts and jeopardizes the health and economic viability of the state’s cattle industry, United States Department of Agriculture’s (USDA) bTB free accreditation status of the state, and the health of wildlife and humans alike. There have been at least seven people that likely contracted bTB from deer in Michigan, United States (12). Thus, persistent spillback transmission of bTB from deer to cattle is of great concern to the livestock industry, governing livestock and wildlife agencies, and the public.

Wildlife managers and local cattle producers have implemented several strategies in their efforts to reduce the incidence of bTB in deer and decrease risks to cattle (13, 14). Mitigation strategies directed at wildlife have included actions such as exclusionary fences (15), increased opportunity for recreational harvest of deer, restrictions on baiting and feeding of deer (16), and issuing disease control permits to landowners and USDA-Wildlife Services (USDA-WS) by the Michigan Department of Natural Resources (MDNR). Though impactful, the apparent prevalence of bTB in the area has remained between 1 and 2% for the last several years (17–19). The stalled reduction in apparent prevalence and continued transmission of bTB from deer to cattle necessitates the need for additional novel management strategies to be integrated with ongoing efforts to aid in combating bTB (14).

Vaccines are a widely used tool proven to safely and effectively protect lives in human and veterinary medicine. They play significant roles in maintaining animal health, enhancing food safety, and reducing the risk of zoonotic disease transmission to humans (20, 21). Vaccines licensed or approved for use in food-producing animals must undergo rigorous regulatory testing and are considered safe for human consumption after a prescribed slaughter withdrawal period due to the degradation of vaccine components in the animal’s body (22). While vaccines are most often administered through injections, oral immunization is required to effectively deliver vaccines to a large proportion of populations of free-ranging wildlife, because trap-vaccinate-release methods are inefficient at a landscape scale (23, 24). The employment of vaccines to combat diseases in wildlife reservoirs is becoming more prominent. The most notable example is the extensive use of oral rabies vaccine in North America and Europe, which has been employed to manage rabies in wildlife for over 40 years (25).

Specific to bTB in wildlife, efforts trialing oral vaccination have demonstrated success with European badgers in Ireland (26, 27), Eurasian wild boar (28) and red deer in Spain (29, 30), and Brushtail possums in New Zealand (31). Thus, a vaccine for bTB delivered to deer has potential to contribute to a decrease in deer-to-deer, deer-to-cattle and deer-to-human transmission of *M. bovis* and advance the eradication effort. *M. bovis* Bacille Calmette-Guérin Danish Strain 1,331 vaccine (hereafter, BCG) is a live attenuated bacteria used as a human vaccine for over 100 years (32) and has received much attention as the most widely researched vaccine candidate for use in wildlife reservoirs (33). Research has demonstrated that vaccination of captive deer with BCG reduces disease severity by decreasing gross lesions, thus suggesting potential for reducing transmission and minimizing endemic infection in wildlife (34, 35). Though this reduced severity of infection demonstrates an immune response and thus a level of protection, it does not mean BCG-vaccinated animals are 100% immune to *M. bovis* infection (34). Relative to food safety, there has been no evidence of BCG persistence in the meat or milk of vaccinated livestock (36, 37), thus the vaccine has a low risk of transmission through consumption of these products. Two additional features of the BCG vaccine are that overdosing of individual animals and non-target species consumption of vaccine are not a concern, as has been demonstrated in studies (33, 38–42) with deer and genera of non-target animals that have potential to consume BCG baits.

Previous successes in delivering food-based baits to deer have demonstrated potential for delivering pharmaceuticals to populations of deer, furthering the concept of oral delivery as a viable tool for vaccination (43–45). Food matrices that mimic seasonal food sources, such as residual agricultural crops, that are made available to deer in late winter to early spring when snow cover is diminished, and deer are food stressed is an effective strategy for reaching targeted deer (45, 46). With progress demonstrating that BCG may be a valuable tool to help eradicate bTB in deer and thus livestock in MI, and in the development of oral delivery strategies, the next step and goal of this current effort is to, for the first time, evaluate the ability to deliver BCG-laden vaccine delivery units (DUs) to free-ranging deer. Knowledge from this effort will highlight the practicality of this vaccination strategy and any challenges that need to be addressed before implementing a broad-scale vaccination program as part of the integrated management effort to combat bTB.

## 2 Materials and methods

### 2.1 Study location

Our study took place in southwestern Alpena County of northeastern lower Michigan, United States from mid-March through April 2024. The area is part of Deer Management Unit 452, where bTB is endemic in deer and the apparent prevalence is the highest (47). Results reported in Feuka et al. (45) informed where within this area we chose to work. Depending on methods used, deer densities ranged from about 10–12 deer/km^2^ (48) or 6–11 deer/km^2^ (18), though historically have been estimated to be up to 18 deer/km^2^ (24). The area is made up primarily of forested and agricultural lands. Conifer stands of northern white cedar (*Thuja occidentalis*) and balsam fir (*Abies balsamea*) occupy the lowlands and provide thermal cover to deer in winter, while uplands consist of aspen (*Populus* spp.), maple (*Acer* spp.) and other deciduous trees (49). Beef and dairy cattle are the primary livestock in the area and bTB has been found in over 80 herds in the last 30 years (50). The main crops produced are hay, soybeans, corn, and wheat. Annual average temperatures are 6.6° C and rain and snow totals average 72.5 cm and 175.0 cm, respectively (51–53). Elevation ranged from 150 to 390 m above sea level (52).

### 2.2 Delivery unit and alginate sphere design

**Figure 1:**
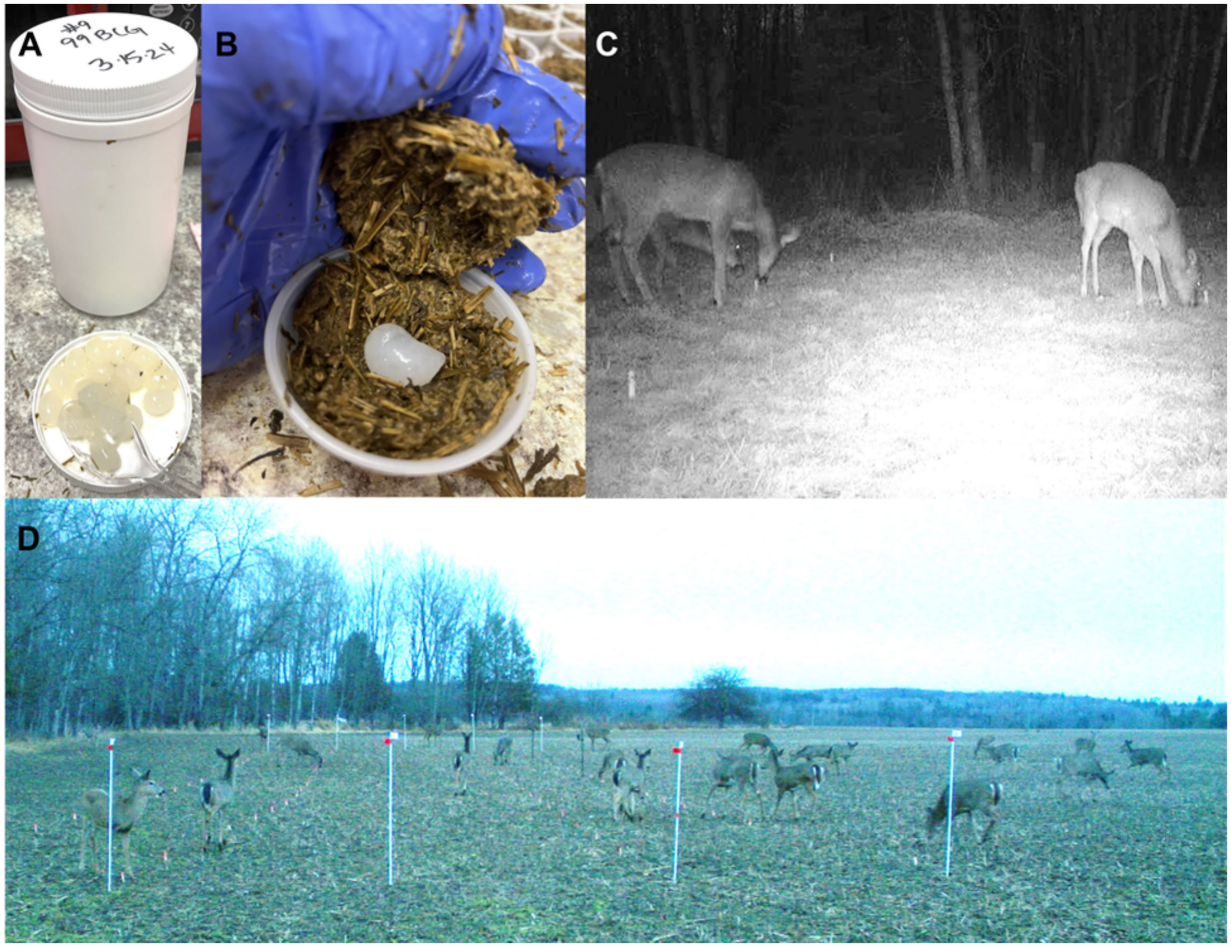
Images of vaccine spheres **(A)**, spheres being incorporated into the food matrix **(B)**, and of deployment from video **(C)** and index **(D)** cameras during a 2024 vaccine deployment against bovine tuberculosis in white-tailed deer (*Odocoileus virginianus*) in northeastern lower Michigan, United States.

The DU matrix recipe used was developed during previous studies (44–46) and further refined in preparation for this evaluation. We prepackaged 6 kg bags of cultured alfalfa (Chaffhaye, Pivotal Feeds, Dell City, TX, United States) and 4 kg bags of vital wheat gluten (Vital Wheat Gluten Flour, Bob’s Red Mill Natural Foods, Milwaukie, OR, United States) to facilitate the efficient production of batches of 100 DUs to be deployed within a day of being prepared. Prior to packaging, the cultured alfalfa was flash-frozen with liquid nitrogen and shredded in a leaf shredder (GUO056 120 V AC, Super Handy, Ontario, CA, United States) until stems were pulverized and finely ground. For each batch, one bag of alfalfa, one bag of gluten, 5 L tap water, and 500 mL of molasses (Black Ops Deer Anthem, Worthington, MN, United States) were combined in a large plastic tub and mixed thoroughly. To prepare each individual DU (placebo or containing vaccine), we began by placing about 15 kernels of whole corn (*Zea mays*) in the bottom of a 60-ml plastic cup (DART Disposable Portion Cup, Amazon.com) for visual appeal. We then compressed ~30 g of the bait matrix into the cup. If the batch of DUs was to contain spheres of vaccine, one sphere was then placed in the cup and gently covered with an additional ~10 g of the matrix, which was compressed around the edges to adhere to underlying matrix material (Figure 1). We then capped the cup with a plastic lid. We estimated the cost of each vaccine DU to be approximately $6.50, not including labor, with about 85% of the cost related to producing the BCG. If scaled up for operational deployment, production efficiencies would be easily achievable and costs per vaccine DU would be much lower.

With seed stock provided by the National Animal Disease Center of the USDA/Agricultural Research Service (Ames, IA, United States), Colorado Serum Company (Denver, CO, United States) produced the liquid BCG (bulk vaccine) at a concentration of 1×10^9^ organisms per ml which was kept frozen until just prior to use. While the vaccine was thawing, we prepared solutions of calcium lactate and alginic acid. We then diluted the vaccine into the calcium lactate solution to a concentration of 5×10^8^ organisms per ml. Alginate spheres were made by pipetting the combined BCG and calcium lactate solution into a bath of alginic acid solution and leaving to react for 10 min, creating gel-like spheres that encapsulated a liquid 2-ml dose of BCG that were designed to burst upon mastication by a deer and bathe the oral cavity and posterior pharynx, including tonsils, while being ingested. Spheres were stored in water and refrigerated at 4°C until use (<10 days). Field use of this unlicensed vaccine was authorized by the USDA/Center for Veterinary Biologics and the Michigan Department of Agriculture and Rural Development.

### 2.3 Plot setup

**Figure 2:**
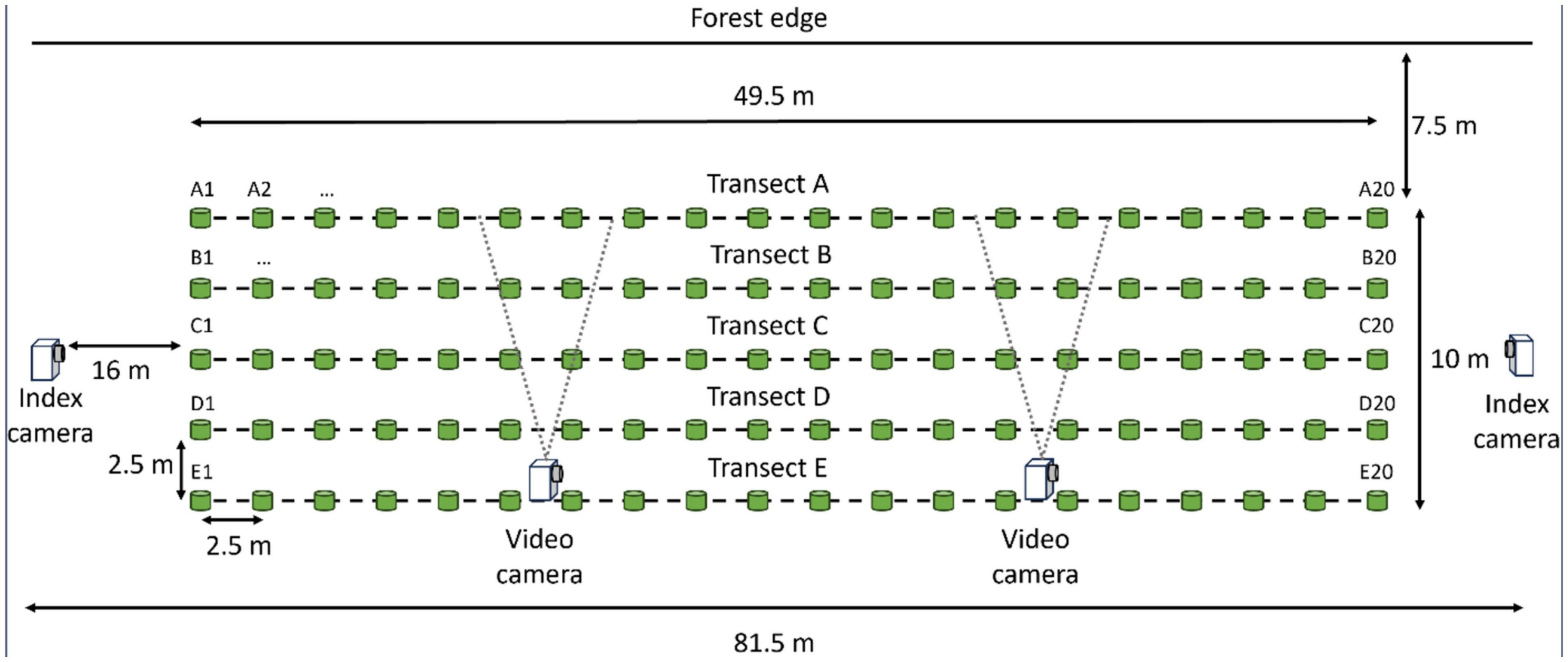
Layout of the 50-m by 10-m, 100 delivery unit grids on agricultural fields next to forest edges during a 2024 vaccine deployment against bovine tuberculosis in white-tailed deer (*Odocoileus virginianus*) in northeastern lower Michigan, United States.

We started monitoring 20 dormant agricultural fields that had been planted in hay, soybeans, corn, or wheat for deer presence in late February 2024. At likely spots along field edges, we dispersed ~3.8 kg of whole corn to entice deer to frequent the area and evaluate the potential of the area for vaccine deployment. We selected the first 11 field sites where we observed reliable use by deer to set up plots and deploy DUs. We established plots 2.5 m from field edges near vegetation types that could provide protective cover for deer. Each plot was 50-m long and 10-m wide, they consisted of five parallel transects 2.5-m apart and running the length of each study plot (Figure 2). Every 2.5-m along each transect, we placed a uniquely numbered, pink wooden marker (6-inch Multi-purpose Sticks, Amazon.com) to denote where to place DUs. To index levels of deer and non-target visitation at each study plot, we installed two “index cameras” (Figure 1; Reconyx, PC900, Holmen, WI, United States) 13.7-m from each end of the study plots. Index cameras were programmed to capture one time-lapse image every 5 min. To characterize contact and consumption of DUs, we installed two “video cameras” (Figure 1; Reconyx, Hyperfire 2 Professional, Holmen, WI, United States) within each study plot that focused on 6 DUs each. We set video cameras to record motion-activated video for 15 s with no quiet period between triggers. Positioning of video cameras within the length of plots was informed by deer sign (e.g., tracks, trails).

### 2.4 Delivery unit deployment and consumption

After setting up study plots, we deployed 100 placebo DUs (consisting of bait matrix only) spaced 2.5-m apart (at each marker) for one or two acclimation nights. The acclimation nights served two purposes: first, they accustomed deer to locating and consuming DUs; and second, they provided baseline data on the number of deer visiting study plots. The number of acclimation nights was influenced by deer presence and placebo DU consumption. In general, if <5 deer were detected in index images or < 25% of VDUs were consumed, we replaced consumed placebo DUs and collected data for an additional night. If consumption was greater than 25% we proceeded to deploy vaccine DUs for two nights. During the day before the first vaccine night, we collected any remaining placebo DUs and deployed 100 vaccine DUs that each contained a sphere of vaccine. The following day we recorded consumed vaccine DUs, did not replace those that were consumed, and left those that remained out for a second night. The next morning, we documented the number of additional missing vaccine DUs. At this time, the treatment for the plot was complete and the site was decommissioned.

Delivery unit consumption by deer was approximated in two ways, by direct visual assessment of presence or absence of DUs at each marker in a plot and by reviewing video footage of the subset of vaccine DUs in the field of view of video cameras. Visually, if a DU was completely absent upon a daily check it was considered to have been completely consumed by a deer or other animal. Delivery units were documented as partially consumed if they had been fed upon, but pieces of alfalfa matrix or alginate sphere were observed at the DU location. Only completely consumed DUs were included in our analyses. Intact DUs were recorded as present.

We used video camera footage to determine the percentage of absent vaccine DUs that were consumed by deer versus being consumed or otherwise removed by non-target vertebrate species and to estimate the probability of any individual animal consuming a vaccine DU given (a) that they appeared to notice it by vision or scent, and (b) that they touched it. For each of these events, we recorded the time, date, site, and species interacting with a vaccine DU.

### 2.5 Statistical analyses

For each plot we looked at images taken from both index cameras, from the time we left a site the day prior until we arrived back the next morning, to find the single image with the most deer in it. The number of deer in this image was used to estimate the absolute minimum number of deer that visited that site that night. We used this number, then, to approximate the maximum number of DUs consumed per deer by dividing the number of fully consumed DUs by the number of deer.

For each site, we tallied how many DUs were fully and partially consumed and summarized data across sites by calculating the mean, median, and standard deviation of consumption values for each trial night. We calculated the proportion of newly consumed DUs by dividing the number of fully consumed DUs by the total number of DUs available at each site each night.

With video data, we calculated the probability of deer consuming a vaccine DU given they noticed it and given they touched it by dividing the number consumed once noticed and once touched. We summarized across sites by calculating the mean, median, and standard error for each night VDUs were deployed.

## 3 Results

### 3.1 DU deployment and consumption

**Figure 3:**
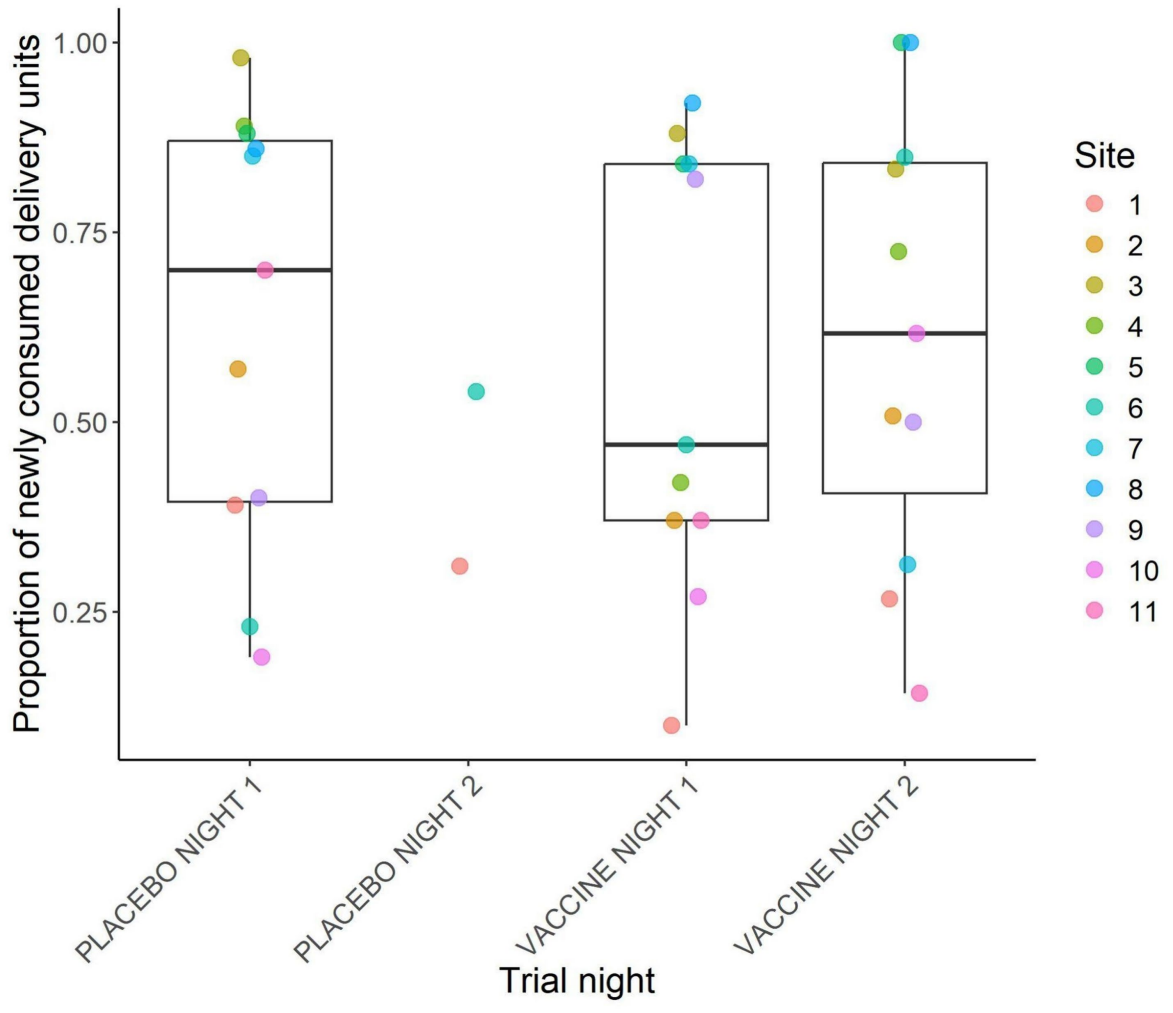
Proportion of newly and fully consumed vaccine delivery units per trial night. Points represent individual site values and boxplots represent summaries across sites. Only sites with data for the entire trial are represented. This was during a 2024 vaccine deployment against bovine tuberculosis in white-tailed deer (*Odocoileus virginianus*) in northeastern lower Michigan, United States.

**Figure 4:**
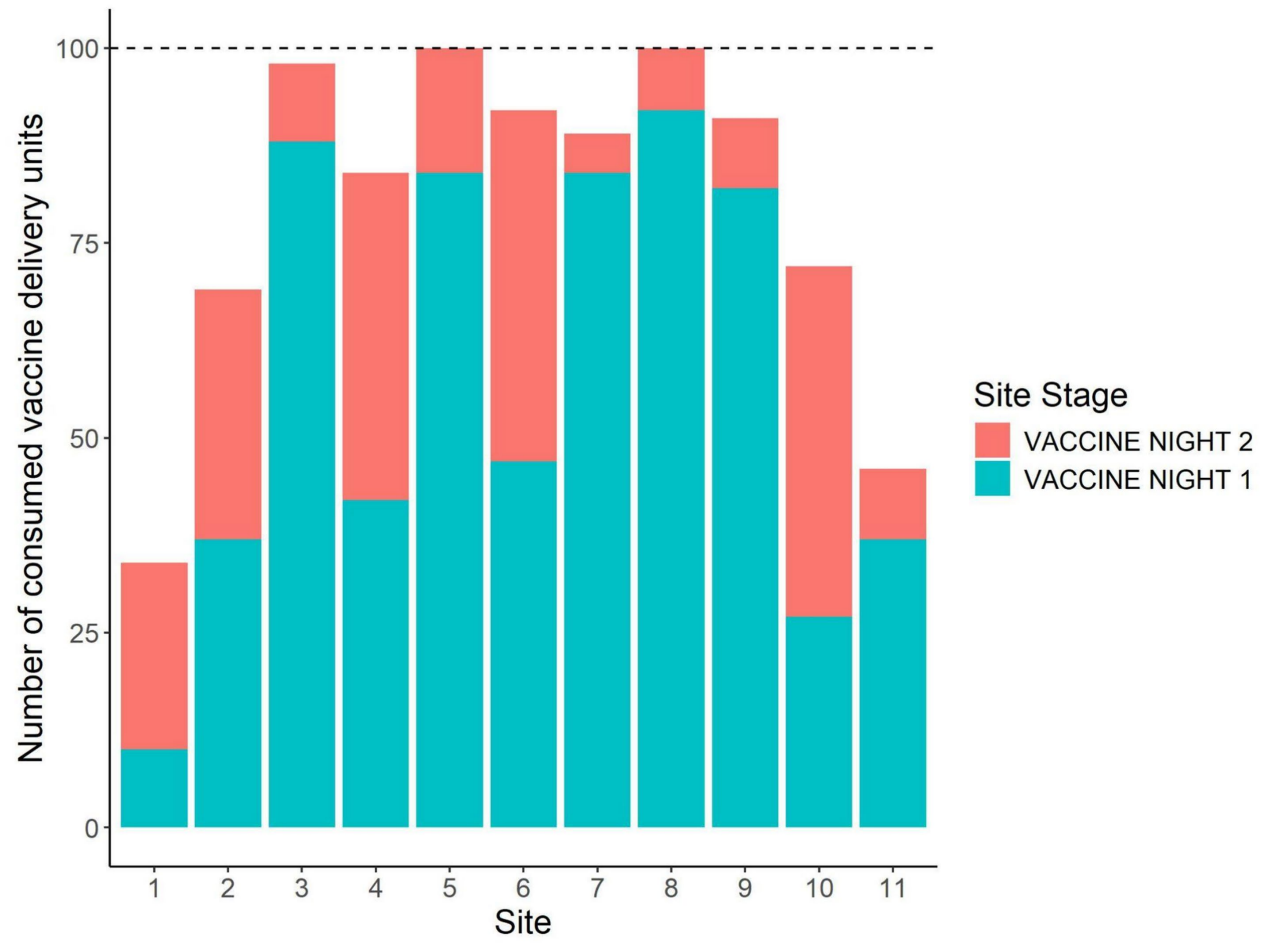
Number of vaccine delivery units consumed on each of the nights BCG vaccine was deployed by site. This was during a 2024 vaccine deployment against bovine tuberculosis in white-tailed deer (*Odocoileus virginianus*) in northeastern lower Michigan, United States.

Across all sites and trial nights, a total of 779 placebo DUs and 875 vaccine DUs were fully consumed, 23 placebo and 125 vaccine DUs were partially consumed, and 497 placebo and 570 vaccine DUs remained where we had placed them. The proportion of DUs consumed by night and site are plotted in Figure 3. Across sites, the mean proportion of DUs consumed was 63.1% (±8.6%) on placebo night 1, 57.3% (±8.8%) on vaccine night 1, and 61.4% (±8.9%) on vaccine night 2. Only two sites had a second placebo night, and they had consumption rates of 31 and 54%. All sites but two had >50% total consumption of vaccine DUs across the two nights of vaccine deployment, and at two sites 100% of vaccine DUs were consumed (Figure 4).

**Figure 5:**
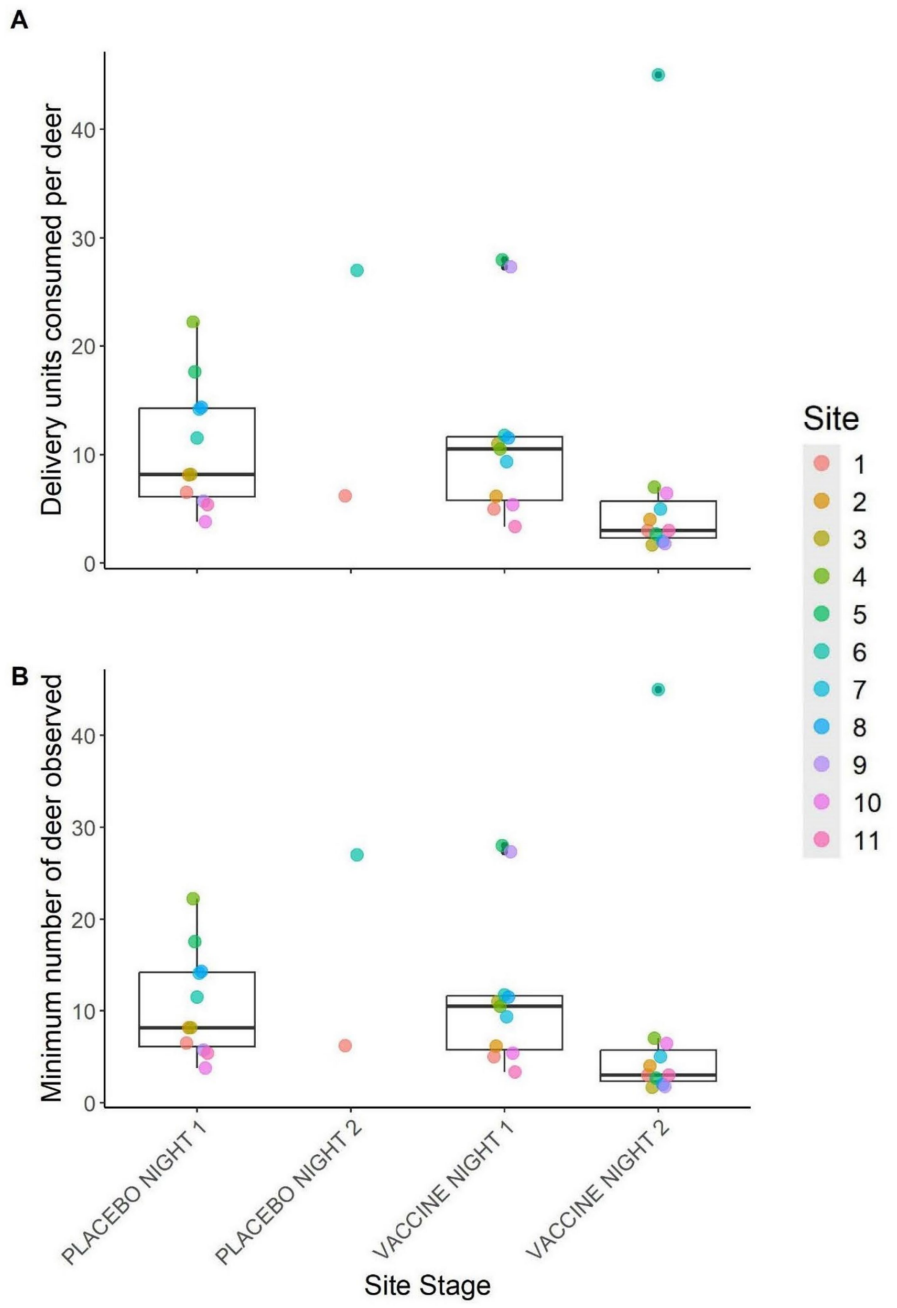
**(A)** Number of vaccine delivery units consumed by white-tailed deer for each trial night and **(B)** the estimated number of deer used to calculate values in **(A)**. Values in **(B)** represent the maximum number of deer observed in a single index camera image per trial night per site. Points represent individual site values and box plots depict the median (inner horizontal line), 25 and 75th percentiles (box borders), and 5 and 95th percentiles (vertical lines). Only sites with data for the entire trial are represented. This was during a 2024 vaccine deployment against bovine tuberculosis in white-tailed deer (*Odocoileus virginianus*) in northeastern lower Michigan, United States.

Summaries from index camera images of minimum numbers of deer visiting sites and thus estimated maximum numbers of DUs consumed per deer are displayed in Figure 5. The number of individually identifiable deer observed at sites ranged from one to 13 per site, per night, and the maximum number of DUs consumed per deer was estimated to be from 1.7 to 45. Across sites, a mean of 10.7 (±1.8) DUs were fully consumed per deer on placebo night 1, 11.8 (±2.5) per deer on vaccine night 1, and 7.4 (±3.8) per deer on vaccine night 2. The two sites that had a second placebo night had nightly consumption of 6.2 and 27 DUs per deer on placebo night 2. All estimates were within one standard error of one another.

We obtained complete data sets from video cameras at seven of the 11 sites on vaccine nights. Of the 434 times we documented vertebrates noticing DUs, 84% of the time (*n* = 365) it was by deer, 11% of the time (*n* = 49) by raccoons (*Procyon lotor*), 3% (*n* = 13) by wild turkeys (*Meleagris gallopavo*), and 2% by Virginia opossum (*Didelphis virginiana*), striped skunk (*Mephitis mephitis*), and gray squirrels (*Sciurus carolinensis*) combined. From the video we observed deer consuming 145 entire vaccine DUs, but just one non-target, a raccoon, was witnessed to consume an entire DU. There were 23 instances where we documented non-targets consuming kernels of corn off DUs (17 raccoons, 4 wild turkeys, and 2 squirrels).

The probability of a deer consuming a vaccine DU that they noticed was 0.11 (±0.04) on vaccine night 1 and 0.19 (±0.06) on vaccine night 2. The probability of consumption was higher if they noticed and then touched a DU, 0.21 (±0.10) on vaccine night 1 and 0.45 (±0.14) on vaccine night 2. The differences between nights differed by at least one standard error for each type of encounter. Anecdotally, each night of a trial, deer became more familiar with DUs and recognized them as a quality food, appearing to search them out and consume them later in a trial.

## 4 Discussion

This effort is the culmination of two long-term, multi-faceted research thrusts; one developing the vaccine in controlled laboratory and pen settings and one developing the attractive, food-based matrix and strategy for concealing the vaccine and placing it optimally on the landscape for wild deer. Foundational vaccine studies include those by Palmer et al. (33, 41, 45, 54, 55) and Nol et al. (56). Another study has demonstrated success protecting penned deer with BCG encapsulated in alginate spheres (Bioggiatto et al., unpublished data) and work to further assess viability of spheres encased in our DU food matrix is planned. These controlled studies provided the necessary background to support evaluating the practicality of oral vaccination of wild deer with BCG. Foundational delivery studies include Palmer et al. (43) who tested an early molasses-based bait while Fischer et al. (44), Dressel (45), and Dressel et al. (46) developed and evaluated strategies for large-scale vaccine delivery in natural field settings. These efforts greatly informed the successful delivery and uptake of vaccine-laden DUs in this study.

We previously reported that 66% of deer in a similarly designed precursor study, with very similar numbers of deer visits per night, consumed DUs containing a biomarker (46). Thus, we expected about the same percentage of deer would consume vaccine DUs during the current study. As minimum numbers of deer visiting sites per night revealed averages of 11.8 and 7.4 DUs were consumed per individual deer on vaccine nights 1 and 2, respectively, many vaccine DUs were wasted due to over consumption because a deer only needs to consume 1 or 2 to be vaccinated. Therefore, going forward, strategies to disperse DUs such that fewer are consumed per individual and thus available to a larger proportion of the deer population within an area need to be developed. For example, as long as consumption is satisfactory relative to the number of deer estimated to be using the area on placebo nights and vaccine night 1, it may not be necessary to deploy vaccine in the same place for a second night. Thus, more DUs would be available to be delivered to different nearby deer thereby increasing the proportion of the local population that consumes vaccine and becomes protected from bTB. The importance of this was demonstrated in Pandey et al. (57), where they found that vaccinating 50% of the deer could lead to an 86% probability of eradicating bTB from the area over a 30-year period, as was similar to findings reported in Ramsey et al. (13). Thus, it will be important to maximize the proportion of deer in the population that consume vaccine DUs to increase the probability of elimination of the disease and reduce the timeline to eradication. Relatedly, there is evidence from pen studies of vaccinated deer shedding BCG to non-vaccinated deer (58–60) which in the wild could lead to a larger proportion of deer in the area where vaccine was deployed becoming protected.

We demonstrated high levels of oral uptake of BCG vaccine DUs by free-ranging deer (Figures 4, 5). Despite abnormally warm and dry winter conditions during this study, it is encouraging that deer visitation and DU consumption was similar to a previous study conducted with rhodamine B biomarker instead of BCG in 2016 (46), during which winter conditions were more typical. Relative to the previous 20 years, daily temperatures were above the normal range most days and accumulated snowfall was ~70% lower than the previous low (2006–2007) (61, 62) These realities provide confidence that our methods could be successfully used in operational management even under relatively warm or dry conditions in late-winter-early spring. Another reason we targeted late winter to offer DUs to deer, besides that deer are most nutritionally stressed at this time of year in northern climates (63), is that many non-target wildlife species that may also be attracted to DUs are least active during this period, only foraging occasionally to conserve energy (64–66). Accordingly, though non-targets may have been more active during our study than during most winters, we still documented little visitation or uptake of DUs by non-targets.

The use of BCG, a live vaccine, merits additional discussion. Though it can be used to vaccinate cattle against bTB, it is not licensed or used in the United States and other countries because it makes it impossible to distinguish between vaccinated animals and those that actually have bTB using current diagnostics (29, 33, 67, 68). The same potential for false positives holds true for deer, which are routinely tested by the MDNR to assess bTB prevalence rates. For this reason, in future research we plan to incorporate a biomarker into our vaccine DUs, thus allowing managers to differentiate between vaccinated and diseased animals while also providing data on the percentage of the deer population that has been vaccinated. Depending on the biomarker, results may then be available to hunters to aid in their personal decision-making regarding consumption. Studies to date in vaccinated deer and wild boar have shown that BCG rarely persists in any tissues longer than 6–9 months post-vaccination. In these studies, BCG has been only detected in lymphoid tissue and intestinal organs, never muscle (33, 41, 55, 56, 69, 70).

## 5 Conclusion

For over 30 years bovine tuberculosis has been known to persist in deer and has spilled back into cattle in Michigan, (United States) (10) which results in social and economic consequences for a variety of stakeholders. Thus, additional means are needed to address the issue. At present, integrated management efforts to control the disease include maintaining restrictions on recreational baiting of deer, encouraging very liberal recreational harvest of deer, broad usage of deer control permits by landowners and agencies, fencing of stored feed and other cattle resources, and other husbandry strategies. The methods employed in this study were effective and scalable for enticing deer to consume DUs. Therefore, vaccination of deer could be considered and implemented into management as an additional measure to potentially reduce transmission of bTB to cattle and among deer to effectively reduce bTB prevalence in the area. Additionally, ongoing efforts are exploring potential applications of oral vaccines for immunizing numerous wildlife species against diseases such as plague, Lyme disease, brucellosis, chronic wasting disease, and pseudorabies (71–76) and we hope they benefit from the advancements we describe here.

## Data Availability

The raw data supporting the conclusions of this article will be made available by the authors, without undue reservation.

## References

[1] Palmer MV. *Mycobacterium bovis*: characteristics of wildlife reservoir hosts. Transbound Emerg Dis. (2013) 60:1–13. doi: 10.1111/tbed.12115, PMID: 24171844

[2] Palmer MV Kanipe C Lombard JE Boggiatto PM. Bovine tuberculosis at the Interface of cattle, wildlife, and humans In: Rezaei N, editor. Tuberculosis: Integrated studies for a complex disease. Cham: Springer International Publishing (2023). 829–46.

[3] Delahay RJ Cheeseman CL Clifton-Hadley RS. Wildlife disease reservoirs: the epidemiology of *Mycobacterium bovis* infection in the European badger (*Meles meles*) and other British mammals. Tuberculosis. (2001) 81:43–9. doi: 10.1054/tube.2000.0266, PMID: 11463223

[4] Payne A Ruette S Jacquier M Richomme C Lesellier S Middleton S. Estimation of bait uptake by badgers, using non-invasive methods, in the perspective of Oral vaccination against bovine tuberculosis in a French infected area. Front Vet Sci. (2022) 9:787932. doi: 10.3389/fvets.2022.787932, PMID: 35359678 PMC8961513

[5] Byrne AW Kenny K Fogarty U O’Keeffe JJ More SJ McGrath G. Spatial and temporal analyses of metrics of tuberculosis infection in badgers (*Meles meles*) from the Republic of Ireland: trends in apparent prevalence. Prev Vet Med. (2015) 122:345–54. doi: 10.1016/j.prevetmed.2015.10.013, PMID: 26556049

[6] Nugent G Buddle B Knowles G. Epidemiology and control of *Mycobacterium bovis* infection in brushtail possums (*Trichosurus vulpecula*), the primary wildlife host of bovine tuberculosis in New Zealand. N Z Vet J. (2015) 63:28–41. doi: 10.1080/00480169.2014.963791, PMID: 25290902 PMC4566891

[7] Wielgus E Caron A Bennitt E De Garine-Wichatitsky M Cain B Fritz H. Inter-group social behavior, contact patterns and risk for pathogen transmission in Cape Buffalo populations. J Wildl Manag. (2021) 85:1574–90. doi: 10.1002/jwmg.22116

[8] Naranjo V Gortazar C Vicente J De La Fuente J. Evidence of the role of European wild boar as a reservoir of *Mycobacterium tuberculosis* complex. Vet Microbiol. (2008) 127:1–9. doi: 10.1016/j.vetmic.2007.10.002, PMID: 18023299

[9] Aranaz A De Juan L Montero N Sánchez C Galka M Delso C. Bovine tuberculosis (*Mycobacterium bovis*) in wildlife in Spain. J Clin Microbiol. (2004) 42:2602–8. doi: 10.1128/JCM.42.6.2602-2608.2004, PMID: 15184440 PMC427808

[10] Schmitt SM Fitzgerald SD Cooley TM Bruning-Fann CS Sullivan L Berry D. Bovine tuberculosis in free-ranging white-tailed deer in Michigan. J Wildl Dis. (1997) 33:749–58. doi: 10.7589/0090-3558-33.4.749, PMID: 9391958

[11] O’Brien DJ Schmitt SM Fitzgerald SD Berry DE Hickling GJ. Managing the wildlife reservoir of *Mycobacterium bovis*: The Michigan, USA, experience. Vet Microbiol. (2006) 112:313–23. doi: 10.1016/j.vetmic.2005.11.014, PMID: 16376030

[12] Sunstrum J. Human disease due to *Mycobacterium bovis* linked to free-ranging deer in Michigan. Clin Infect Dis. (2024) 78:637–45. doi: 10.1093/cid/ciae009, PMID: 38207126

[13] Ramsey DSL O’brien DJ Cosgrove MK. Forecasting eradication of bovine tuberculosis in Michigan white-tailed deer. J Wildl Manag. (2014) 78:240–54. doi: 10.1002/jwmg.656

[14] Ver Cauteren KC Lavelle MJ Campa H. Persistent spillback of bovine tuberculosis from White-tailed deer to cattle in Michigan, USA: status, strategies, and needs. Front Vet Sci. (2018) 5:301. doi: 10.3389/fvets.2018.00301, PMID: 30555834 PMC6281989

[15] Lavelle MJ Kay SL Pepin KM Grear DA Campa H Ver Cauteren KC. Evaluating wildlife-cattle contact rates to improve the understanding of dynamics of bovine tuberculosis transmission in Michigan, USA. Prev Vet Med. (2016) 135:28–36. doi: 10.1016/j.prevetmed.2016.10.009, PMID: 27931926

[16] Cosgrove MK O’Brien DJ Ramsey DSL. Baiting and feeding revisited: modeling factors influencing transmission of tuberculosis among deer and to cattle. Front Vet Sci. (2018) 5:306. doi: 10.3389/fvets.2018.00306, PMID: 30564585 PMC6288431

[17] Okafor C Grooms DL Bruning-Fan CS Averill JJ Kaneene JB. Descriptive epidemiology of bovine tuberculosis in Michigan (1975–2010): lessons learned. Vet Med Int. (2011) 2011:874924:1–13. doi: 10.4061/2011/874924, PMID: 21776355 PMC3135262

[18] Feuka A Pandey A Cosgrove M Moriarty M Duffiney A Campa H. Estimating landscape spread of a low-prevalence disease using multiple surveillance methods. J Appl Ecol. (2024) 61:575–87. doi: 10.1111/1365-2664.14574

[19] O’Brien DJ Kao RR Little RA Enticott G Riley SJ. The road not traveled: Bovine tuberculosis in England, Wales, and Michigan, USA. One Health Cases. (2023):ohcs20230028. doi: 10.1079/onehealthcases.2023.0028

[20] Pastoret P-P. Role of vaccination in animal health. Bull Académie Natl Médecine. (2012) 196:589–90. doi: 10.1016/S0001-4079(19)31794-7 23472348

[21] Laxminarayan R Gleason A Sheen J Saad-Roy CM Metcalf CJ Palmer GH. Unlock the potential of vaccines in food-producing animals. Science. (2024) 384:1409–11. doi: 10.1126/science.adj5918, PMID: 38935731

[22] Clarke I Walker J Hennessy D Kreeger J Nappier J Crane J. Inherent food safety of a synthetic gonadotropin-releasing factor (GnRF) vaccine for the control of boar taint in entire male pigs, Int J Appl Res Vet Med. (2008) 6.

[23] Bastille-Rousseau G Gorman NT McClure KM Nituch L Buchanan T Chipman RB. Assessing the efficiency of local rabies vaccination strategies for raccoons (*Procyon lotor*) in an urban setting. J Wildl Dis. (2024) 60:26–38. doi: 10.7589/JWD-D-23-00059, PMID: 37924240

[24] Cosgrove MK Campa H Schmitt SM Marks DR Wilson AS O’Brien DJ. Live-trapping and bovine tuberculosis testing of free-ranging white-tailed deer for targeted removal. Wildl Res. (2012) 39:104. doi: 10.1071/WR11147

[25] Rupprecht CE Buchanan T Cliquet F King R Müller T Yakobson B. A global perspective on Oral vaccination of wildlife against rabies. J Wildl Dis. (2024) 60:241–84. doi: 10.7589/JWD-D-23-00078, PMID: 38381612

[26] Gormley E Ní D Fitzsimons T O’Keeffe J McGrath G Madden JM. Protective immunity against tuberculosis in a free-living badger population vaccinated orally with *Mycobacterium bovis* Bacille Calmette–Guérin. Transbound Emerg Dis. (2022) 69:e10–9. doi: 10.1111/tbed.14254, PMID: 34331741

[27] Gormley E Corner LAL. Pathogenesis of *Mycobacterium bovis* infection: the badger model as a paradigm for understanding tuberculosis in animals. Front Vet Sci. (2018) 4:247. doi: 10.3389/fvets.2017.00247, PMID: 29379792 PMC5775213

[28] Díez-Delgado I Sevilla IA Romero B Tanner E Barasona JA White AR. Impact of piglet oral vaccination against tuberculosis in endemic free-ranging wild boar populations. Prev Vet Med. (2018) 155:11–20. doi: 10.1016/j.prevetmed.2018.04.002, PMID: 29786520

[29] Thomas J Risalde MÁ Serrano M Sevilla I Geijo M Ortíz JA. The response of red deer to oral administration of heat-inactivated *Mycobacterium bovis* and challenge with a field strain. Vet Microbiol. (2017) 208:195–202. doi: 10.1016/j.vetmic.2017.08.007, PMID: 28888638

[30] Martinez-Guijosa J Casades-Marti L González-Barrio D Aranaz A Fierro Y Gortázar C. Tuning oral-bait delivery strategies for red deer in Mediterranean ecosystems. Eur J Wildl Res. (2020) 66:51. doi: 10.1007/s10344-020-01389-8

[31] Nugent G Yockney IJ Whitford EJ Cross ML Aldwell FE Buddle BM. Field trial of an aerially-distributed tuberculosis vaccine in a low-density wildlife population of Brushtail possums (*Trichosurus vulpecula*). PLoS One. (2016) 11:e0167144. doi: 10.1371/journal.pone.0167144, PMID: 27893793 PMC5125682

[32] Fine PEM The BCG. Story: lessons from the past and implications for the future. Clin Infect Dis. (1989) 11:S353–9. doi: 10.1093/clinids/11.Supplement_2.S353, PMID: 2652252

[33] Palmer MV Thacker TC. Use of the human vaccine, *Mycobacterium bovis* Bacillus Calmette Guérin in deer. Front Vet Sci. (2018) 5:244. doi: 10.3389/fvets.2018.00244, PMID: 30349823 PMC6186790

[34] Nol P Palmer MV Waters WR Aldwell FE Buddle BM Triantis JM. Efficacy of oral and parenteral routes of Mycobacterium bovis bacille Calmette-Guerin vaccination against experimental bovine tuberculosis in white-tailed deer (Odocoileus virginianus): a feasibility study. J Wildl Dis. (2008) 44:247–59. doi: 10.7589/0090-3558-44.2.247, PMID: 18436658

[35] Palmer MV Thacker TC Waters WR Robbe-Austerman S. Oral vaccination of White-tailed deer (*Odocoileus virginianus*) with *Mycobacterium bovis* Bacillus Calmette-Guerin (BCG). PLoS One. (2014) 9:e97031. doi: 10.1371/journal.pone.0097031, PMID: 24804678 PMC4013142

[36] Williams GA Scott-Baird E Núñez A Salguero FJ Wood E Houghton S. The safety of BCG vaccination in cattle: results from good laboratory practice safety studies in calves and lactating cows. Heliyon. (2022) 8:e12356. doi: 10.1016/j.heliyon.2022.e12356, PMID: 36590473 PMC9800532

[37] Pérez De Val B Vidal E López-Soria S Marco A Cervera Z Martín M. Assessment of safety and interferon gamma responses of *Mycobacterium bovis* BCG vaccine in goat kids and milking goats. Vaccine. (2016) 34:881–6. doi: 10.1016/j.vaccine.2016.01.004, PMID: 26795364

[38] Hayes FM Haring CM Traum J. Vaccination of swine against tuberculosis with Calmette-Guérin culture. BCG Hilgardia. (1932) 7:235–61. doi: 10.3733/hilg.v07n06p235

[39] Aldwell FE Keen DL Parlane NA Skinner MA De Lisle GW Buddle BM. Oral vaccination with *Mycobacterium bovis* BCG in a lipid formulation induces resistance to pulmonary tuberculosis in brushtail possums. Vaccine. (2003) 22:70–6. doi: 10.1016/S0264-410X(03)00539-5, PMID: 14604573

[40] Aldwell FE Tucker IG De Lisle GW Buddle BM. Oral delivery of *Mycobacterium bovis* BCG in a lipid formulation induces resistance to pulmonary tuberculosis in mice. Infect Immun. (2003) 71:101–8. doi: 10.1128/IAI.71.1.101-108.2003, PMID: 12496154 PMC143408

[41] Palmer MV Thacker TC Waters WR Robbe-Austerman S Aldwell FE. Persistence of *Mycobacterium bovis* bacillus Calmette-Guérin (BCG) Danish in white-tailed deer (*Odocoileus virginianus*) vaccinated with a lipid-formulated oral vaccine. Transbound Emerg Dis. (2014) 61:266–72. doi: 10.1111/tbed.12032, PMID: 23173832

[42] Gormley E Ní Bhuachalla D O’Keeffe J Murphy D Aldwell FE Fitzsimons T. Oral vaccination of free-living badgers (*Meles meles*) with Bacille Calmette Guérin (BCG) vaccine confers protection against tuberculosis. PLoS One. (2017) 12:e0168851. doi: 10.1371/journal.pone.0168851, PMID: 28121981 PMC5266210

[43] Palmer MV Stafne MR Waters WR Thacker TC Phillips GE. Testing a molasses-based bait for oral vaccination of white-tailed deer (*Odocoileus virginianus*) against *Mycobacterium bovis*. Eur J Wildl Res. (2014) 60:265–70. doi: 10.1007/s10344-013-0777-9

[44] Fischer JW Blass CR Walter WD Anderson CW Lavelle MJ Hall WH. Evaluating a strategy to deliver vaccine to white-tailed deer at a landscape level: delivery of vaccine to deer. Wildl Soc Bull. (2016) 40:394–9. doi: 10.1002/wsb.635

[45] Dressel D. Development of strategies to orally deliver vaccine for bovine tuberculosis to white-tailed deer of northeastern lower Michigan. East Lansing, Michigan, USA: Michigan State University (2017).

[46] Dressel D VerCauteren KC Lavelle MJ Snow NP Campa H. Use of rhodamine B as a biomarker in a simulated oral vaccine deployment against bovine tuberculosis in white-tailed deer. Front Vet Sci. (2024) 11:1354772. doi: 10.3389/fvets.2024.1354772, PMID: 38414651 PMC10896993

[47] Carstensen M O’Brien DJ Schmitt SM. Public acceptance as a determinant of management strategies for bovine tuberculosis in free-ranging U.S. wildlife. Vet Microbiol. (2011) 151:200–4. doi: 10.1016/j.vetmic.2011.02.046, PMID: 21439739

[48] O’Brien DJ Schmitt SM Fitzgerald SD Berry DE. Management of bovine tuberculosis in Michigan wildlife: current status and near term prospects. Vet Microbiol. (2011) 151:179–87. doi: 10.1016/j.vetmic.2011.02.042, PMID: 21414734

[49] Felix AB Campa H Millenbah KF Winterstein SR Moritz WE. Development of landscape-scale habitat-potential models for forest wildlife planning and management. Wildl Soc Bull. (2004) 32:795–806. doi: 10.2193/0091-7648(2004)032[0795:DOLHMF]2.0.CO;2

[50] MDARD. Bovine tuberculosis eradication program quarterly update in: Development MDoAaR. Animal Industry Division Michigan Department of Agriculture and Rural Development (MDARD). (2023).

[51] Knapp BD. Soil survey of Presque Isle County. Michigan: USDA Soil Conservation Service (1988). 252 p.

[52] Williams TE. Soil survey of Alcona County. Michigan: Natural Resources Conservation Service (1998).

[53] Sitar KL. Seasonal movements, habitat use patterns, and population dynamics of white-tailed deer (*Odocoileus virginianus*) in an agricultural region of northern lower Michigan. East Lansing, Michigan, USA: Michigan State University (1996).

[54] Palmer MV Waters WR Thacker TC. Vaccination of white-tailed deer (*Odocoileus virginianus*) with *Mycobacterium bovis* bacille Calmette-Guérin (BCG) results in positive tuberculin skin test results in a dose-dependent fashion. Res Vet Sci. (2020) 129:70–3. doi: 10.1016/j.rvsc.2020.01.010, PMID: 31954316

[55] Palmer MV Thacker TC Waters WR Robbe-Austerman S Lebepe-Mazur SM Harris NB. Persistence of *Mycobacterium bovis* Bacillus Calmette-Guérin in White-tailed deer (*Odocoileus Virginianus*) after Oral or parenteral vaccination. Zoonoses Public Health. (2010) 57:e206–e212. doi: 10.1111/j.1863-2378.2010.01329.x, PMID: 20707863

[56] Nol P Rhyan JC Robbe-Austerman S McCollum MP Rigg TD Saklou NT. The potential for transmission of BCG from orally vaccinated White-tailed deer (*Odocoileus virginianus*) to cattle (*Bos taurus*) through a contaminated environment: experimental findings. PLoS One. (2013) 8:e60257. doi: 10.1371/journal.pone.0060257, PMID: 23565211 PMC3615014

[57] Pandey A Feuka AB Cosgrove M Moriarty M Duffiney A VerCauteren KC. Wildlife vaccination strategies for eliminating bovine tuberculosis white-tailed deer populations. PLoS Comput Biol. (2024) 20:e1011287. doi: 10.1371/journal.pcbi.1011287 38175850 PMC10793927

[58] Palmer MV Thacker TC Waters WR. Vaccination with *Mycobacterium bovis* BCG strains Danish and Pasteur in White-tailed deer (*Odocoileus virginianus*) experimentally challenged with *Mycobacterium bovis*. Zoonoses Public Health. (2009) 56:243–51. doi: 10.1111/j.1863-2378.2008.01198.x, PMID: 19175569

[59] Palmer MV Thacker TC Waters WR. Vaccination of white-tailed deer (*Odocoileus virginianus*) with *Mycobacterium bovis* bacillus Calmette Guerín. Vaccine. (2007) 25:6589–97. doi: 10.1016/j.vaccine.2007.06.056, PMID: 17688976

[60] Palmer M Thacker T Waters WR Robbe-Austerman S. Investigations on deer to deer and deer to cattle transmission of the vaccine *Mycobacterium bovis* Bacillus Calmette-Guérin (BCG). J Vaccines Vaccin. (2010) 1:1–5. doi: 10.4172/2157-7560.1000104

[61] National Weather Service. Annual snowfall for Gaylord, MI [2006-2007]. National Oceanic and Atmospheric Administration. (2007). Available at: https://www.weather.gov/apx/snow [Accessed September 9, 2024]

[62] National Weather Service. Climate data for Gaylord, MI [02/24-04/24]. National Oceanic and Atmospheric Administration. (2024). Available at: https://www.weather.gov/wrh/Climate?wfo=apx [Accessed September 9, 2024]

[63] Mautz W. Sledding on a bushy hillside: The fat cycle in deer. Wildl Soc Bull. (1978) 6:88–90.

[64] Kanda LL Fuller TK Friedland KD. Temperature sensor evaluation of opossum winter activity. Wildl Soc Bull. (2005) 33:1425–31. doi: 10.2193/0091-7648(2005)33[1425:TSEOOW]2.0.CO;2

[65] Zeveloff SI. Raccoons: a natural history. Smithsonian Institution. (2013):209.

[66] Mutch GRP Aleksiuk M. Ecological aspects of winter dormancy in the striped skunk (*Mephitis mephitis*). Can J Zool. (1977) 55:607–15. doi: 10.1139/z77-077

[67] López V González-Barrio D Lima-Barbero JF Ortiz JA Domínguez L Juste R. Oral administration of heat-inactivated *Mycobacterium bovis* reduces the response of farmed red deer to avian and bovine tuberculin. Vet Immunol Immunopathol. (2016) 172:21–5. doi: 10.1016/j.vetimm.2016.03.003, PMID: 27032499

[68] Balseiro A Thomas J Gortázar C Risalde MA. Development and challenges in animal tuberculosis vaccination. Pathogens. (2020) 9:472. doi: 10.3390/pathogens9060472, PMID: 32549360 PMC7350370

[69] Beltrán-Beck B Romero B Sevilla IA Barasona JA Garrido JM González-Barrio D. Assessment of an Oral *Mycobacterium bovis* BCG vaccine and an Inactivated *M. bovis* preparation for wild boar in terms of adverse reactions, vaccine strain survival, and uptake by nontarget species. Clin Vaccine Immunol. (2014) 21:12–20. doi: 10.1128/CVI.00488-13, PMID: 24173022 PMC3910919

[70] Buddle BM Vordermeier HM Chambers MA De Klerk-Lorist L-M. Efficacy and safety of BCG vaccine for control of tuberculosis in domestic livestock and wildlife. Front Vet Sci. (2018) 5:259. doi: 10.3389/fvets.2018.00259, PMID: 30417002 PMC6214331

[71] Stafford KC Williams SC Van Oosterwijk JG Linske MA Zatechka S Richer LM. Field evaluation of a novel oral reservoir-targeted vaccine against *Borrelia burgdorferi* utilizing an inactivated whole-cell bacterial antigen expression vehicle. Exp Appl Acarol. (2020) 80:257–68. doi: 10.1007/s10493-019-00458-1, PMID: 31898760

[72] Richer LM Brisson D Melo R Ostfeld RS Zeidner N Gomes-Solecki M. Reservoir targeted vaccine against *Borrelia burgdorferi*: a new strategy to prevent Lyme disease transmission. J Infect Dis. (2014) 209:1972–80. doi: 10.1093/infdis/jiu005, PMID: 24523510 PMC4038139

[73] Rocke TE Tripp DW Russell RE Abbott RC Richgels KLD Matchett MR. Sylvatic plague vaccine partially protects prairie dogs (*Cynomys* spp.) in field trials. EcoHealth. (2017) 14:438–50. doi: 10.1007/s10393-017-1253-x, PMID: 28643091 PMC5662665

[74] Kazemi-Roudsari M Doosti A Jami M-S. Design of an oral vaccine using *Lactococcus lactis* against brucellosis: an in vitro and in vivo study. AMB Express. (2024) 14:2. doi: 10.1186/s13568-023-01638-4, PMID: 38170414 PMC10764709

[75] Napper S Schatzl HM. Oral vaccination as a potential strategy to manage chronic wasting disease in wild cervid populations. Front Immunol. (2023) 14:1156451. doi: 10.3389/fimmu.2023.1156451, PMID: 37122761 PMC10140515

[76] Maresch C Lange E Teifke JP Fuchs W Klupp B Müller T. Oral immunization of wild boar and domestic pigs with attenuated live vaccine protects against pseudorabies virus infection. Vet Microbiol. (2012) 161:20–5. doi: 10.1016/j.vetmic.2012.07.002, PMID: 22832373

